# Reference Interval for Non-HDL-Cholesterol, Remnant Cholesterol and Other Lipid Parameters in the Southern Iranian Population; Findings From Bandare Kong and Fasa Cohort Studies

**DOI:** 10.34172/aim.2024.03

**Published:** 2024-01-01

**Authors:** Mojtaba Farjam, Masoumeh Kheirandish, Amin Ghanbarnejad, Amin Reza Nikpoor, Abdolazim Nejatizadeh, Teymour Aghamolaei, Mehdi Shahmoradi, Hesam Alizade, Reza Homayounfar, Hassan Zarei, Sahar Ghavidel, Vahide Jamshidi, Ebrahim Eftekhar

**Affiliations:** ^1^Noncommunicable Diseases Research Center, Fasa University of Medical Sciences, Fasa, Iran; ^2^Endocrinology and Metabolism Research Center, Hormozgan University of Medical Sciences, Bandar Abbas, Iran; ^3^Social Determinants in Health Promotion Research Center, Faculty of Health, Hormozgan University of Medical Sciences, Bandar Abbas, Iran; ^4^Molecular Medicine Research Center, Hormozgan Health Institute, Hormozgan University of Medical Sciences, Bandar Abbas, Iran; ^5^Cardiovascular Research Center, Hormozgan University of Medical Sciences, Bandar Abbas, Iran; ^6^Infectious and Tropical Diseases Research Center, Hormozgan Health Institute, Hormozgan University of Medical Sciences, Bandar Abbas, Iran; ^7^National Nutrition and Food Technology Research Center, Shahid Beheshti University of Medical Sciences, Tehran, Iran

**Keywords:** Lipid profile, Non-HDL Cholesterol, Reference interval, Remnant cholesterol

## Abstract

**Background:**

Growing evidence shows the undisputable role of non-HDL-C and remnant cholesterol (remnant-C) in cardiovascular disease (CVD) risk assessment and treatment. However, the reference interval (RI) for these lipid parameters is not readily available. The aim of the present investigation was to determine the age and sex-specific RIs for non-HDL-C and remnant-C as well as other lipid parameters among a healthy population in southern Iran. We also report the RI of lipid parameters in rural and urban residents, smokers and post-menopausal women.

**Methods:**

Among 14063 participants of Bandare Kong and Fasa cohort studies, 792 healthy subjects (205 men and 578 women) aged 35-70 years were selected. Fasting blood samples were used for determination of total cholesterol (TC), triglycerides (TG) and HDL-C using colorimetric methods. Non-HDL-C and remnant-C were calculated using the valid formula. The 2.5th and 97.5th percentiles were calculated and considered as RI.

**Results:**

In the total population (n=792, age 35-70), RIs for non-HDL-C and remnant-C was 74.0-206.8 and 8.0-52.7 mg/dL, respectively. Age (35-44 and≥45 years) and gender-specific RIs for serum non-HDL-C and remnant-C were determined. Remnant-C and non-HDL-C level were different between sex and age categories. The mean value of all lipid parameters except HDL-C was higher in men, urban residents, subject with age≥45 years and smokers.

**Conclusion:**

This is the first study in which the RIs for non-HDL-C and remnant-C in southern Iran are reported. This may help physicians to conveniently use these lipid parameters for patient care and better cardiovascular risk assessment.

## Introduction

 Dyslipidemia, by definition, is elevated total cholesterol (TC), triglycerides (TG), low-density lipoprotein cholesterol (LDL-C), and or reduced serum high-density lipoprotein cholesterol (HDL-C) level. It is considered as one of the important modifiable risk factors for cardiovascular disease (CVD).^1^ CVD is the leading cause of mortality in the world. It is responsible for 38–50% of mortality among the Iranian population.^2^

 Over the past decade, the tendency to use non-HDL-C and remnant cholesterol (remnant-C) as a risk factor for CVD has increased greatly. It has been shown that non-HDL-C is a superior marker of CVD risk than LDL-C, especially in subjects with high serum triglycerides concentration. Additionally, levels of remnant-C have been proposed to partially explain residual risk not obtained with LDL-C.^3^ Significant associations of remnant-C with cardiovascular events have been shown.^4,5^

 In a prospective investigation, individuals with high non-HDL-C levels were at increased CVD risk independent of LDL-C levels.^6^ Therefore, in recent guidelines, there is a lot of emphasis on considering non-HDL-C as the second target of treatment.^3,7,8^ Non-HDL-C can be calculated as TC minus HDL-C and remnant-C can be obtained using this formula: (TC) –(HDL-C) – (LDL-C).

 Dyslipidemia is affected by a variety of factors such as diet, lifestyle habits and genetic predisposition. For this reason, the Adult Treatment Panel III committee has defined the reference interval (RI) for plasma lipids to reduce the risk of CVD.^9^ The RIs are different over the world base on the variable race, ethnicity, environmental, and lifestyle factors. Thus, population-based studies are the most reliable and appropriate way to determine the RIs.^10^ Considering the different conditions in each population, previous studies have determined their RI for objectively establishing abnormal lipid profiles.^11,12^

 As HDL-C, LDL-C and TC are measured as routine lipid profile, the necessary data to calculate non-HDL-C and remnant-C is easily available with no additional cost. One of the limitations of the laboratories for non-HDL-C and remnant-C report is lack of a valid RI. To the best of our knowledge, the adult RIs for non-HDL-C and remnant-C were established only in one of the previous studies.^13^ No study has reported the RIs of serum non-HDL-C and remnant-C in the Iranian adult population. Therefore, this study was conducted to provide the RIs for non-HDL-C, remnant-C and other lipid parameters according to different age and gender categories among the healthy middle-aged and elderly population in southern Iran. In addition, the RIs for all lipid indices in cigarette and hookah smokers and menopausal women were reported.

## Materials and Methods

###  Study Population

 We examined the Bandare Kong and Fasa cohort study participants (14 063 people), the first phase of the Prospective Epidemiological Research Studies in IrAN (PERSIAN) that has been previously described in detail.^14^ All 14 063 study participants were from southern Iran, including 4063 subjects from the Bandar Kong cohort of the Hormozgan province and 10 000 individuals from the Fasa cohort of the Fars province. After applying the exclusion criteria, a total of 792 apparently healthy individuals including 403 participants from Bandare Kong cohort and 389 participants from Fasa cohort were considered for final analysis. From this selected population, 205 (25.8%) were men and 587 (74.2%) were women.

 The age of participants was 35-70 years, their BMI was 18-25 kg/m^2^ and they were recruited between November 17, 2016, and November 22, 2018. The participants were excluded if they had the following conditions: BMI ≥ 25 Kg/m^2^, lactating or pregnant women, smokers, diabetes, hypertension, dyslipidemia, CVD, liver and kidney disorders, malignancy, and thyroid disease. Those who consumed alcohol, drugs such as calcium channel blockers, oral contraceptive drugs, diuretics, thyroid and cardiac drugs, antihypertensive agents, steroids, and hormone replacement therapy were also excluded.

###  Determination of Non-HDL-C, Remnant-C and Other Lipid Profile Reference Intervals

 The non-HDL-C, remnant-C and other lipid profile RIs were determined based on the age and sex categories as well as the place of residence (urban or rural regions). Furthermore, the RI of this lipid parameters was reported in a group of healthy cigarette-smokers (n = 133) and hookah-smokers (n = 129) and menopausal women (n = 251). The criteria for entering the study were the same as those mentioned above, except that the people in these groups were smokers or postmenopausal.

 The sociodemographic and anthropometric data were collected by trained interviewers and a face-to-face interview. Age, gender, place of residence and menopause status were recorded. Weight was measured using a digital scale (measurement accuracy of 0.5 kg) while wearing the least amount of clothing. Height was measured with subjects standing shoeless with their shoulders set normally. BMI was calculated as weight in kilograms divided by the square of the person’s height in meters. Blood pressure was measured with a standard mercury sphygmomanometer. The thresholds for defining lipid disorders were those suggested by the ATP III guidelines.^15^ Cigarette and hookah smoking status was based on self-report data. Cigarette smoker was defined by current smoking and having smoked at least 100 cigarettes in lifetime.^16^ Menopause was defined as permanent cessation of menstruation. Menstruation time was considered retrospectively; i.e. 1 year without menses.^17^

 Blood samples were collected into 6-mL vacuum-tubes with a clot activator (Vacutest, Italy). The subjects fasted for 10-12 hours before sample collection, and the time of sampling was from 8:00 AM to 10:00 AM. Blood samples were allowed to clot for 30 minutes at room temperature and then centrifuged at 1000 g for 10 minutes. Serum was separated and aliquoted into 0.5-mL microtubes and stored at -80 ºC until analysis. All lipid parameters were measured on the chemistry autoanalyzer BT1500 (Biotechnical Instruments, Rome, Italy) using commercially standard kits (Pars Azmoon, Tehran, Iran). TG level was assessed by the enzymatic GPO-PAP (lipase/glycerol kinase) method, whereas TC was determined by the CHOD-PAP (cholesterol oxidase) method. For HDL-C assay, other lipoproteins including LDL, VLDL, and chylomicron were blocked by anti-human β-lipoprotein antibodies, and then the cholesterol content of the HDL particles was measured using the CHOD-PAP method. LDL-C was calculated by Friedwald equation (LDL-C = TC–HDL-C – TG/5). A direct method was used to determine serum LDL-C for those individuals with TG more than 200 mg/dL. Very low-density lipoprotein-cholesterol (VLDL-C), non-HDL-C, and remnant-C were estimated using the following formula: VLDL-C = TG/5, non-HDL-C = TC – HDL-C, remnant-C = non-HDL-C – LDL-C.^18^

 Test quality control was done every day using commercial serum control samples (Pars Azmoon kit, Tehran, Iran) at two different levels of normal (level 1) and pathologic (level 2) for each analyte. The Westgard-multi-rule algorithm was used to interpret the results of serum control samples. The results had to be within the desirable limits before the subject’s serum testing. For each analyte, standard deviations (SDs) were calculated, and the percent coefficient of variation (%CV) was presented in Table 1.

**Table 1:** The Results of Coefficient of Variation (CV) of Lipid Profile in Normal (Level 1) and Pathologic (Level 2) Serum Control Samples

**Lipid Profile**	**Reference Mean of Serum Control**		**Laboratory Mean of Serum Control**		**%CV**	
	**Level I**	**Level II**	**Level I**	**Level II**	**Level I**	**Level II**
TG (mg/dL)	97.0	222.0	92.0	228.0	3.2	2.8
TC (mg/dL)	144.0	190.0	149.6	194.4	2.9	2.3
HDL-C (mg/dL)	42.5	-	49.1	-	2.0	-
LDL-C (mg/dL)	73.8	123.0	71.5	126.8	2.4	2.3

CV, coefficient of variation; HDL-C, high-density lipoprotein cholesterol; LDL-C, low-density lipoprotein cholesterol; TC, total cholesterol; TG, triglyceride.

###  Statistical Analysis

 Outliers were deleted from the dataset using Dixon outlier range statistics as suggested by the Clinical and Laboratory Standards Institute (CLSI).^19^ In Dixon’s test, D is defined as the absolute difference between the most extreme and the next extreme, and R is the range; if the D/R ratio exceeds 1/3, the extreme value is considered an outlier and should be discarded. Statistical analysis was performed using the IBM SPSS software (SPSS Inc., Chicago, IL, version 20). All parameters were shown as mean ± SD, median, percentile 2.5%, percentile 97.5%, lower limit of 95% confidence interval (CI), and upper limit of 95% CI. Since most of the biochemical parameters did not follow a Gaussian distribution, the non-parametric methodology was used for determination of RIs, as recommended by the International Federation of Clinical Chemistry (IFCC).^20^

## Results

 In this study, a total of 792 participants who were classified as healthy people according to the criteria were enrolled. Of these, 205 were male with an average age of 48.54 ± 9.61 years and 587 were female with an average age of 48.51 ± 9.47 years.

 RIs (2.5 percentile and 97.5 percentile) for serum non-HDL-C, remnant-C and routine lipid profile for the total population as well as for male and female subjects are illustrated in Table 2. As indicated, all mean lipid values were higher in males; however, HDL-C was higher in the female gender (51.87 ± 11.09 mg/dL) compared to male participants (45.72 ± 8.74 mg/dL). As shown in Table 2, the mean concentration of non-HDL-C in males (151.56 ± 33.0 mg/dL) was higher than females (127.25 ± 32.36 mg/dL) and the RI value was different between males and females. The mean level of remnant-C was also higher in males (25.84 ± 15.42 mg/dL) compared to females (18.92 ± 9.14 mg/dL) and a different RI was reported for males and females. The histograms of lipid profile among healthy individuals are shown in Figure S1 (see Supplementary file 1).

**Table 2:** Reference Intervals for Non-HDL-C, Remnant-C and Other Lipid Indices According to Sex of the Study Population

		**TC (mg/dL)**	**TG (mg/dL)**	**HDL-C (mg/dL)**	**LDL-C (mg/dL)**	**VLDL-C (mg/dL)**	**LDL-C/ HDL-C**	**TG/HDL-C**	**Non-HDL-C (mg/dL)**	**Remnant-C (mg/dL)**	**TC/HDL Ratio**
Total (n = 792)	Mean ± SD	184.26 ± 34.84	98.17 ± 44.43	50.22 ± 10.85	111.46 ± 30.25	19.63 ± 8.88	2.34 ± 0.76	2.09 ± 1.19	133.74 ± 34.42	20.75 ± 11.55	3.80 ± 1
	Median	182.6	87	49	110.7	17.4	2.2826	1.7502	132	17.52	3.6875
	Percentile 2.5% (95% CI)	122.6 (119.2-126.1)	39.7 (35.2-44.1)	32 (30.9-33.1)	55.6 (52.5-58.6)	7.94 (7.0-8.8)	1.0 (0.9-1.1)	0.7 (0.5-0.8)	74 (70.5-77.4)	8 (6.8-9.1)	2.1 (2.1-2.3)
	Percentile 95% (95% CI)	260 (256.5-263.4)	217.3 (212.8-221.8)	76.4 (75.3-77.5)	176.5 (173.4-179.5)	43.47 (42.5-44.3)	4 (3.9-4.0)	5.4 (5.2-5.5)	206.8 (203.3-210.2)	52.7 (51.6-53.9)	6.0 (5.9-6.12)
Male (n = 205)	Mean ± SD	197.18 ± 34.11	115.10 ± 49.72	45.72 ± 8.74	124.76 ± 27.81	23.02 ± 9.94	2.83 ± 0.76	2.63 ± 1.44	151.56 ± 33.01	25.84 ± 15.42	4.46 ± 1.10
	Median	194.00	102.00	45.00	124.00	20.40	2.78	2.28	151.00	21.50	4.31
	Percentile 2.5% (95% CI)	134.1 (127.4-140.8)	49.0 (38.9-59.0)	31.0 (29.2-32.7)	71.1 (65.6-76.6)	9.8 (7.7-11.8)	1.4 (1.3-1.6)	0.9 (0.6-1.2)	89.3 (82.8-95.7)	10.0 (6.9-13.0)	2.73 (2.5-2.9)
	Percentile 95% (95% CI)	265.5 (258.8-272.2)	244.0 (233.9-254.0)	63.0 (61.2-64.7)	181.9 (176.4-187.4)	48.8 (46.7-50.8)	4.1 (3.9-4.2)	6.63 (6.3-6.9)	214.7 (208.2-221.1)	70.0 (66.9-73.0)	7.02 (6.8-7.2)
Female (n = 587)	Mean ± SD	179.75 ± 33.98	92.61 ± 41.10	51.87 ± 11.09	106.89 ± 29.72	18.52 ± 8.22	2.18 ± 0.70	1.91 ± 1.04	127.25 ± 32.62	18.92 ± 9.14	3.57 ± 0.85
	Median	177.10	81.50	50.70	105.26	16.30	2.13	1.64	125.60	16.25	3.50
	Percentile 2.5% (95% CI)	121.4 (117.5-125.4)	38.1 (33.4-42.9)	34.0 (32.7-35.3)	53.7 (50.2-57.1)	7.6 (6.6-8.5)	0.89 (0.8-0.9)	0.69 (0.5-0.8)	72.0 (68.2-75.9)	8.00 (6.92-9.08)	2.17 (2.0-2.2)
	Percentile 95% (95% CI)	253.1 (249.2-257.1)	207.3 (202.5-212.0)	80.6 (79.2-81.9)	172.0 (168.6-175.4)	41.4 (40.5-42.4)	3.7 (3.62-3.79)	4.88 (4.7-5.0)	199.8 (195.9-203.7)	42.7 (41.6-43.8)	5.5 (5.4-5.6)

TC, total cholesterol; TG, triglyceride; HDL-C, high-density lipoprotein cholesterol; LDL-C, low-density lipoprotein cholesterol; VLDL-C, very-low density lipoprotein cholesterol; Remnant-C, remnant-cholesterol.

 As indicated in Table 3, according to age, the participants were divided into two group of 35–44 years old (n = 545) and ≥ 45 years old (n = 247). As seen in Table 3, all lipid profile indices in healthy individuals increased with age, except for the HDL-C level which was not affected by age.

**Table 3:** Reference Intervals for Non-HDL-C, Remnant-C and Other Lipid Indices According to Age of the Study Population

		**TC (mg/dL)**	**TG (mg/dL)**	**HDL-C (mg/dL)**	**LDL-C (mg/dL)**	**VLDL-C (mg/dL)**	**LDL-c/ HDL-c**	**TG/HDL-C**	**Non- HDL-C (mg/dL)**	**Remnant-C (mg/dL)**	**TC/HDL Ratio**
Age: 35-44 years (n = 545)	Mean ± SD	180.96 ± 34.18	94.35 ± 43.1.2	50.14 ± 11.07	108.33 ± 29.66	18.87 ± 8.62	2.29 ± 0.76	2.02 ± 1.17	130.28 ± 33.85	20.26 ± 11.92	3.75 ± 1.00
	Median	180.00	82.50	49.00	107.83	16.50	2.25	1.70	129.35	16.96	3.66
	Percentile 2.5% (95% CI)	122.1 (117.9-126.2)	38.2 (33.0-43.4)	32.0 (30.6-33.3)	53.55 (49.98-57.12)	7.65 (6.60-8.70)	1.01 (0.91-1.10)	0.70 (0.55-0.85)	73.03 (68.87-77.18)	8.00 (6.53-9.47)	2.18 (2.05-2.30)
	Percentile 95% (95% CI)	259.3 (255.2-263.4)	209.1 (203.8-214.3)	78.4 (77.1-79.8)	175.3 (171.8-178.9)	41.8 (40.7-42.8)	4.0 (3.9-4.0)	5.2 (5.1-5.4)	206.0 (201.8-210.1)	60.0 (58.5-61.4)	6.1 (5.9-6.2)
Age > 45 years (n = 247)	Mean ± SD	191.54 ± 35.25	106.44 ± 46.16	50.43 ± 10.41	118.38 ± 30.46	21.29 ± 9.23	2.47 ± 0.78	2.25 ± 1.24	141.21 ± 34.54	21.82 ± 10.66	3.94 ± 1.00
	Median	188.80	96.40	50.00	118.00	19.28	2.38	1.95	138.70	19.06	3.85
	Percentile 2.5% (95% CI)	124.4 (118.1-130.7)	43.7 (35.4-51.9)	32.1 (30.3-34.0)	62.6 (57.1-68.1)	8.7 (7.0-10.3)	1.0 (0.9-1.2)	0.8 (0.6-1.0)	74.9 (68.6-81.1)	9.0 (7.0-10.9)	2.2 (2.1-2.4)
	Percentile 95% (95% CI)	260.8 (254.5-267.1)	229.1 (220.8-237.3)	74.5 (72.6-76.3)	177.0 (171.5-182.4)	45.8 (44.1-47.4)	4.0 (3.9-4.2)	5.5 (5.3-5.8)	208.3 (202.1-214.5)	48.9 (46.9-50.8)	5.9 (5.7-6.1)

TC, total cholesterol; TG, triglyceride; HDL-C, high-density lipoprotein cholesterol; LDL-C, low-density lipoprotein cholesterol; VLDL-C, very low-density lipoprotein cholesterol; Remnant-C, remnant-cholesterol.

 The mean concentration of non-HDL-C in individuals aged 45 years and older (141.21 ± 34.54 mg/dL) was higher than those aged 33-44 years (130.28 ± 33.85 mg/dL). Figure S2 shows the distribution of lipid profile with error bars in healthy people based on sex and age group.

 Another factor studied in the present work was the effect of urban or rural residence of the participants on their lipid profile indices. As shown in Table 4, urban and rural residence was reported for 388 and 404 of the participants, respectively. As seen in Table 4, the average of all lipid profile indices, except for HDL-C, was higher in urban residents than in the rural population. The HDL-C level was 48.41 ± 10.29 mg/dL in urban residents and 52.09 ± 11.12 mg/dL in rural residents. As shown in Table 4, higher non-HDL-C concentration was noted in urban (143.78 ± 33.83 mg/dL) than rural residents (123.52 ± 31.97 mg/dL).

**Table 4:** Reference Intervals for Non-HDL-C, Remnant-C and Other Lipid Indices According to Place of Residence of the Study Population.

		**TC (mg/dL)**	**TG (mg/dL)**	**HDL-C (mg/dL)**	**LDL-C (mg/dL)**	**VLDL-C (mg/dL)**	**LDL-C / HDL-C**	**TG/HDL-C**	**Non- HDL-C (mg/dL)**	**Remnant-C (mg/dL)**	**TC/HDL Ratio**
Urban (n = 387)	Mean ± SD	192.04 ± 34.96	103.20 ± 47.70	48.41 ± 10.29	121.14 ± 27.99	20.64 ± 9.54	2.61 ± 0.77	2.27 ± 1.33	143.78 ± 33.83	22.02 ± 12.94	4.13 ± 1.06
	Median	191.25	91.00	47.00	121.00	18.20	2.58	1.92	143.00	18.00	4.02
	Percentile 2.5% (95% CI)	125.4 (120.4-130.4)	40.0 (33.1-46.8)	31.9 (30.5-33.4)	69.0 (65.0-73.0)	8.0 (6.6-9.3)	1.3 (1.2-1.4)	0.7 (0.5-0.9)	82.0 (77.1-86.8)	8.0 (6.1-9.8)	2.5 (2.4-2.7)
	Percentile 95% (95% CI)	263.5 (258.5-268.5)	235.8 (228.9-242.6)	71.3 (69.8-72.7)	180.2 (176.2-184.2)	47.1 (45.7-48.5)	4.0 (3.0 -4.2)	5.8 (5.6-5.9)	212.3 (207.4-217.1)	61.0 (59.1-62.8)	6.8 (6.6-6.9)
Rural (n = 404)	Mean ± SD	176.79 ± 33.08	93.37 ± 40.55	52.09 ± 11.12	102.26 ± 29.47	18.67 ± 8.11	2.08 ± 0.67	1.92 ± 1.01	123.52 ± 31.97	19.47 ± 9.80	3.49 ± 0.82
	Median	174.00	83.30	50.70	99.82	16.66	2.07	1.68	120.75	16.80	3.42
	Percentile 2.5% (95% CI)	122.1 (117.4-126.7)	39.0 (33.3-44.7)	34.4 (32.8-36.0)	45.8 (41.7-50.0)	7.8 (6.6-8.9)	0.8 (0.7-0.9)	0.7 (0.5-0.8)	68.5 (63.9-73.1)	8.0 (6.6-9.4)	2.0 (1.9-2.1)
	Percentile 95% (95% CI)	250.6 (245.9-255.2)	210.4 (204.7-216.1)	81.0 (79.4-82.6)	161.0 (156.9-165.2)	42.0 (40.9-43.2)	3.5 (3.4-3.6)	4.8 (4.7-4.9)	192.4 (187.8-197.0)	45.1 (43.7-46.5)	5.3 (5.2-5.4)

TC, total cholesterol; TG, triglyceride; HDL-C, high-density lipoprotein cholesterol; LDL-C, low-density lipoprotein cholesterol; VLDL-C, very low-density lipoprotein cholesterol; Remnant-C, remnant-cholesterol.

Table 5 compares the lipid values between healthy cigarette- and hookah-smokers. As the table shows, cigarette smokers had higher lipid values than hookah users, while the HDL-C value was nearly the same. There was a higher mean for all lipid values in cigarette and hookah smokers, except for HDL-C, compared to total study participants (Table 2).

**Table 5:** Reference Intervals for Non-HDL-C, Remnant-C and Other Lipid Indices According to Cigarette and Hookah Use of the Study Population.

		**TC (mg/dL)**	**TG (mg/dL)**	**HDL-C (mg/dL)**	**LDL-C (mg/dL)**	**VLDL-C (mg/dL)**	**LDL-C/ HDL-C**	**TG/HDL-C**	**Non- HDL-C (mg/dL)**	**Remnant-C (mg/dL)**	**TC/HDL Ratio**
Healthy cigarette smokers (n = 133)	Mean ± SD	198.82 ± 36.28	122.33 ± 50.52	44.78 ± 10.51	125.50 ± 27.83	24.47 ± 10.10	2.94 ± 0.83	2.91 ± 1.54	154.22 ± 35.43	28.34 ± 16.34	4.65 ± 1.20
	Median	200.00	110.50	45.00	128.00	22.10	3.07	2.61	154.80	23.00	4.58
	Percentile 2.5% (95% CI)	119.8 (110.9-128.6)	50.4 (37.7-63.2)	27.0 (24.4-29.5)	57.4 (50.6-64.1)	10.1 (7.5-12.6)	1.3 (1.1-1.5)	0.8 (0.4-1.2)	74.4 (65.7-83.0)	10.2 (6.2-14.2)	2.8 (2.5-3.1)
	Percentile 95% (95% CI)	275.6 (266.8-284.4)	254.2 (241.5-267.0)	70.6 (68.1-73.1)	175.9 (169.1-182.6)	50.8 (48.3-53.4)	4.4 (4.2-4.6)	6.6 (6.2-7.0)	225.6 (217.0-234.2)	77.0 (73.0-81.0)	7.7 (7.4-8.0)
Healthy Hookah smokers (n = 129)	Mean ± SD	191.22 ± 34.82	114.64 ± 54.76	45.55 ± 9.62	120.79 ± 27.88	22.93 ± 10.95	2.76 ± 0.79	2.72 ± 1.69	145.60 ± 33.39	24.11 ± 12.89	4.37 ± 1.12
	Median	190.00	101.95	45.00	120.00	20.39	2.74	2.20	143.50	21.00	4.28
	Percentile 2.5% (95% CI)	120.0 (111.3-128.6)	46.2 (32.6-59.8)	26.2 (23.9-28.6)	62.5 (55.6-69.5)	9.2 (6.5-11.9)	1.4 (1.2-1.6)	0.8 (0.4-1.2)	77.3 (69.0-85.6)	9.0 (5.7-12.2)	2.6 (2.3-2.9)
	Percentile 95% (95% CI)	258.0 (249.3-266.6)	233.0 (219.4-246.6)	67.9 (65.5-70.2)	177.9 (171.0-184.8)	46.6 (43.8-49.3)	4.6 (4.4-4.7)	6.6 (6.2-7.10	210.0 (201.7-218.2)	60.4 (57.1-63.60	6.7 (6.4-6.9)

TC, total cholesterol; TG, triglyceride; HDL-C, high-density lipoprotein cholesterol; LDL-C, low-density lipoprotein cholesterol; VLDL-C, very low-density lipoprotein cholesterol; Remnant-C, remnant-cholesterol.

 Finally, Table S1 reports the lipid profile indices of 259 menopausal women. When healthy premenopausal women (female subjects in Table 2) and healthy menopausal women (Supplementary file 1, Table S1) were compared, it was observed that all lipid parameters were in higher range in menopausal women. The mean serum non-HDL-C concentrations in premenopausal and menopausal subjects were 127.25 ± 32.62 mg/dL and 148.97 ± 35.88 mg/dL, and for remnant-C, the values were 18.92 ± 9.14 mg/dL and 24.24 ± 11.53 mg/dL, respectively.

## Discussion

 Non-HDL-C represents the sum of cholesterol content of all atherogenic lipoproteins including LDL, VLDL, intermediate density lipoprotein (IDL), Lipoprotein (a) (Lp(a)), chylomicrons and their remnants.^21^ In recent years, growing evidence shows the importance of non-HDL-C and remnant-C for CVD risk assessment. Interestingly, non-HDL-C has become the second treatment target in many guidelines.^21^ One problem is that reference values for serum non-HDL-C and remnant-C are not available, and laboratories tend to use RIs from the literature. However, the commutability of such data is not accepted.^22^ Therefore, it is necessary to constantly determine and validate the reference range for these lipid parameters. In the present investigation, we report the age- and sex-specific reference values as the 2.5 and 97.5 percentiles for non-HDL-C and remnant-C as well as other lipid parameters including TC, TG, HDL-C, LDL-C, VLDL-C, TG/HDL-C, TC/HDL-C and LDL-C/HDL-C ratios among the 35-70-year-old healthy population in southern Iran.

 According to our findings (Table 2), there is a sex difference for non-HDL-C and remnant-C RIs with higher values pertaining to men. So, it is reasonable to consider gender-specific RIs for non-HDL-C and remnant-C. Similar to our results, Ridefelt et alreported different RIs for non-HDL-C and remnant-C in adult male and female subjects.^13^ It should be noted that the RIs for these two important lipid indices have not been reported in Iranian adult people yet. In the present study, other lipid parameters also showed higher values in men than women, except for HDL-C. This is in agreement with other investigations.^12,23^ Given the different effects of sex hormones on lipid and lipoprotein components, this observation could be ultimately construed. There are significant sex differences in lipid and lipoprotein metabolism that contribute to sex differences. The mechanisms are complex, depending on hormonal effects on tissue and gene-mediated consequences.^24^

 Our results demonstrate a clear increase in the RI of non-HDL-C with age, which is in accordance with previous report.^13^ Therefore, it is necessary to consider age-specific RIs for non-HDL-C. The RIs of some lipid parameters including TC, TG, VLDL-C and LDL-C also increased with age. However, the RI of remnant-C did not change with increasing age (Table 3).

 The lipid reference values in the current study were compared to the Iranian and non-Iranian studies. A study by Azizi et al showed lower median values in TC, LDL-C and higher values in HDL-C than the current study among both males and females, but in terms of median TG values, our female and male population had higher and lower values than the Tehran population, respectively.^25^ There was discrepancy between our values and a study in Ahvaz that presented the values of lipid indices according to gender and ethnicity categories (Lur, Arab and Persian).^26^ Although these studies have been conducted in Iran, the different values could be explained by counting different percentile and standard deviation of values, as well as different population age, ethnicity, and lifestyle.

 There are studies being performed to determine lipid reference values all over the world.^27-34^ The 2.5^th^ and 97.5^th^ or 5^th^ and 95^th^ percentiles based on gender are shown in Table S2. According to Table S2, the lower and upper limits of reference values for TC, TG and LDL-C in the United States^27^ and Canada^28^ were higher and for HDL-C, the RIs were nearly equal compared to the current study. On the other hand, Asian countries including India^11,29,30^ had lower amounts, except for HDL-C, compared to our populations.

 In agreement with the Tehran study,^25^ the upper limits of TC (2657.5 mg/dL in men and 253.1 mg/dL in women), and TG (244.0 mg/dL in men and 207.3 mg/dL in women) were higher, and the lower limits of HDL-C (31.0 mg/dL in men and 34.0 mg/dL in women) were lower than cut-off points of dyslipidemia defined according to the National Cholesterol Education Program Adult Treatment Panel III (NCEP-ATP III) guidelines. The mentioned upper limit values of TC were higher than high risk cut-off points according to the NCEP-ATP III recommendation; however, the upper limit values of LDL-C (181.9 mg/dL in men and 172.0 mg/dL in women) were lower than the high-risk category for cardiovascular incidents.^35^

 Our study showed that median, mean ± SD and reference values of non-HDL-C, remnant-C, TC, TG, LDL-C and all lipid ratios were higher, while HDL-C levels were lower in urban compared to rural residents (Table 4). Potential explanations for the differences in blood lipids of urban and rural dwellers may be attributed to socioeconomic status, unhealthy diet and less physical activity.^36^

 Our study revealed that all lipid profile median and mean values in healthy cigarette- and hookah-smokers (Table 5) were higher than the healthy non-smoker individuals (total population in Table 1). On the other hand, smokers had lower HDL-C compared to their counterparts. Surprisingly, all lipid profile values, except for HDL-C, showed higher mean values in cigarette smokers compared to hookah users. We did not find any study regarding lipid reference values in smokers. Smoking is considered as a significant risk factor for CVD. It is associated with lipid abnormality that is more atherogenic. Numerous studies have consistently found that smoking increases TC, TG, LDL-C and decreases HDL-C level.^37^ However, others showed that smoking reduces LDL-C, HDL-C, TC and increases TG.^38^ In comparison, some studies reported a slightly different smoking effect on lipid profile.^39^ It can be concluded that it is important to define the lipid RIs in the smoker population.

 In the current study for the first time, RIs were reported for non-HDL-C and remnant-C in healthy post-menopausal women (Table S1). Serum non-HDL-C and remnant-C had a wider RI in post-menopausal as compared to premenopausal women (female subjects in Table 1); therefore, it is reasonable to consider different RIs for these two groups of women. Other lipid indices had a higher level in post-menopausal compared to premenopausal women (female subjects in Table 2). In agreement with our results, in a previous investigation on an Iranian population, higher levels of TC, TG and LDL-C, but not HDL-C, were reported in postmenopausal than premenopausal women.^40^ It is clear that determining the reference values needs at least a sample size of 120.^41^ However, the mentioned study included a smaller sample size of post-menopausal women than our study (34 vs. 259). Menopause is defined as ovarian follicular inactivity, which leads to permanent cession of the menstruation period. Various hormonal changes occur in women after menopause, which lead to lipid metabolism alterations and increased coronary heart disease (CAD) risk in women, up to 50 years. CAD incidence is lower in women, but the incidence becomes similar in men and post-menopausal women.^42^

## Strengths and Limitations of the Study

 The current work has several strengths. To the best of our knowledge, this the first investigation in which the reference values for non-HDL-C and remnant-C in a healthy adult Iranian population was determined. In addition, RIs were reported for all lipid parameters in a large sample size of healthy post-menopausal women. Besides, the reference values for lipid profile were determined for the first time for healthy cigarette- and hookah-smokers.

 Our study has some limitations. We could not account for ethnicity, dietary habits and physical activity that might have influenced serum lipid values. Additionally, the population of the present study were selected from southern Iran and may not be representative of the Iranian population in general.

## Conclusion

 In the present study, the age- and sex-specific RIs were reported for non-HDL-C and remnant-C as well as other lipid indices in a healthy cohort population from southern Iran. This helps laboratories to introduce non-HDL-C and remnant-C reference values in test reports and help physicians to conveniently use these lipid parameters for patient care and better cardiovascular risk assessment in southern Iran.

## Supplementary Files

Supplementary file 1 contains Figures S1-S2 and Tables S1-S2.
