# Supplementary material for: Reference Interval for Non-HDL-Cholesterol, Remnant Cholesterol and Other Lipid Parameters in the Southern Iranian Population; Findings From Bandare Kong and Fasa Cohort Studies
Source: Arch Iran Med. 2024 Jan 1;27(1):15–22. doi: 10.34172/aim.2024.03 (PMC10915932; doi:10.34172/aim.2024.03)
Supplement: Supplementary file 1 — contains Figures S1-S2 and Tables S1-S2. [file aim-27-15-s001.pdf]

Arch Iran Med. January 2024;27(1):15-S1.
10.34172/aim.2024.03

Table S1. Reference Intervals for Non-HDL-C, Remnant-C and Other Lipid Indices in Healthy Menopausal Women.

		TC (mg/dL)	TG (mg/dL)	HDL-C (mg/dL)	LDL-C (mg/dL)	VLDL-C (mg/dL)	LDL-C/ HDL-C	TG/HDL -C	Non-HDL-C (mg/dL)	Remnant-C (mg/dL)	TC/HDL Ratio
Healthy Menopausal women (n=259)	Mean ± SD	203.35±37.15	117.22±48.15	52.97±11.64	122.81±31.05	23.44±9.63	2.45±0.79	2.38±1.18	148.97±35.88	24.24±11.53	3.97±0.98
	Median	203.50	110.00	51.60	125.17	22.00	2.35	2.22	149.00	22.00	3.86
	Percentile 2.5% (95% CI)	132.8(126.3-139.2)	51.1(42.8-59.4)	35.0(33.0-37.0)	60.1(54.6-65.5)	10.2(8.5-11.9)	0.9(0.8-1.1)	0.8(0.6-1.0)	82.0(75.6-88.5)	10.0(7.9-12.1)	2.2(2.0-2.4)
	Percentile 95% (95% CI)	276.5(270.0-282.9)	233.1(224.8-241.4)	81.3(79.2-83.3)	182.0(176.5-187.4)	46.6(44.9-48.2)	4.0(3.9-4.1)	5.4(5.1-5.6)	222.6(216.1-229.0)	55.1(53.0-57.2)	6.1(5.9-6.2)

TC, total cholesterol; TG, triglyceride; HDL-C, high density lipoprotein cholesterol; LDL-C, low density lipoprotein cholesterol; VLDL-C, very low density lipoprotein cholesterol; Remnant-C, remnant-cholesterol.

Table S2. Serum Lipid Profile Reference Values in the Present Study and Some Other Studies Over the World.

Gender	Country (reference)	N	population age (year)	TC (mg/dL)	TG (mg/dL)	HDL-C (mg/dL)	LDL-C (mg/dL)
Men	India[11]	865	20-60	23.7–235.1	47.8–205.5	32.1–58.0	63.0–157.8
	Tehran (Iran)[25]	548	>20	121.0–261.0	46.9–301.2	30.9–71.9	54.1–175.2
	Ahvaz (Iran)[26]	460	15-55	178.7–233.4	132.6–258.6	38.3–56.8	112.5–175.7
	USA[27]	4572	20-74	143.8–290.8	54.9–311.8	29.0–68.0	80.0–208.0
	Canada[28]	8348	18-74	118.7–282.7	91.2–394.1	34.42–69.2	54.1–194.9
	India[29]	1161	20-70	121.0–235.1	57.6–262.2	27.8–59.9	61.1–257.2
	India[30]	840	20-80	96.7–234.0	40.7–225.0	24.0–73. 1	41.0–172.8
	Japan[31]	677	4-95	136.1–232.0	40.7–148.8	27.0–77.7	NR
	Finland[32]	292	>30	63.6–326.0	46.0–248.9	30.9–74.2	103.6–254.4
	Burkina Faso[33]	139	15-50	113.3–224.7	-	25.1–68.4	45.6–154.3
	Netherlands[34]	54065	2-49	174.0–223.9	71.7–144.4	19.0–25.1	47.2–67.7
	**Southern Iran (present study)**	**205**	**35-70**	**134.1-265.5**	**49.0-244.0**	**31.0-63.0**	**71.1-181.9**

Women	India[11]	662	20-60	125.7–232.8	47.8–203.7	32.9–65.0	64.2–155.8
	Tehran (Iran)[25]	594	>20	166.3–199.9	38.1–184.2	36.0–83.9	49.9–160.9
	Ahvaz (Iran)[26]	336	15-55	175.8–220.7	106.0–203.4	47.6–71.2	107.0–173.0
	USA[27]	4117	20-74	143.1–305.1	48.7–263.1	32.9–79.7	80.8–220.0
	Canada[28]	8571	18-74	114.1–282.7	31.9–276.4	28.2–80.8	48.0–190.3
	India[29]	762	20-70	119.1–235.1	54.0–217.9	32.1–67.0	56.8–157.0
	India[30]	645	20-80	92.8–263.0	39.9–256.0	22.8–73.1	39.8–172.8
	Japan[31]	468	4-95	135.0–230.9	31.0–134.6	36.0–82.0	NR
	Finland[32]	299	>30	158.9–332.2	40.7–202.0	36.7–85.1	97.1–253.3
	Burkina Faso[33]	140	15-50	113.3–226.6	-	30.6–65.0	44.9–161.7
	Netherland[34]	79475	2-49	113.3–226.6	57.6–105.4	21.7–31.7	42.5–62.3
	**Southern Iran (present study)**	**578**	**35-70**	**121.4-253.1**	**38.1-207.3**	**34.0-80.6**	**53.7-172.0**

NR=not reported

TC, total cholesterol; TG, triglyceride; HDL-C, high density lipoprotein cholesterol; LDL-C, low density lipoprotein cholesterol.

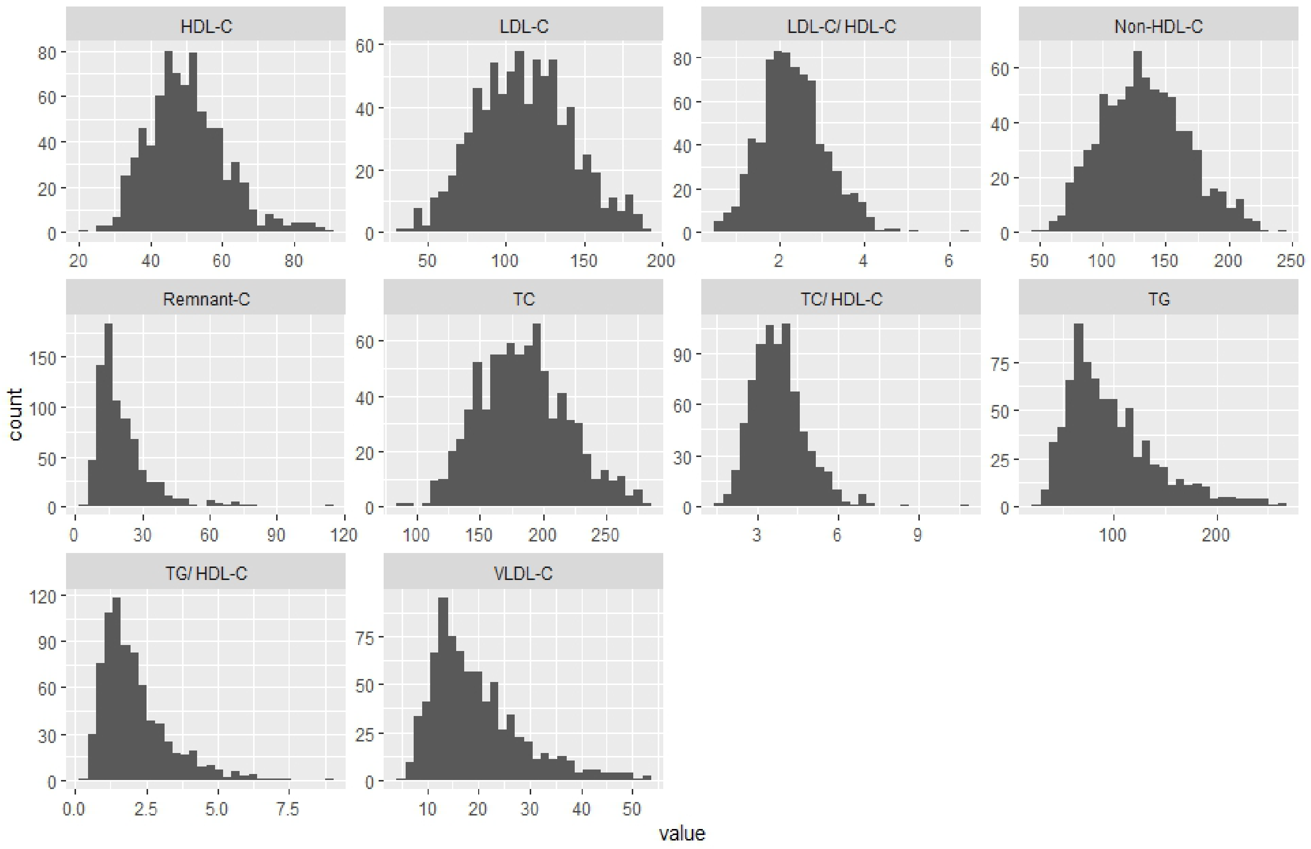

Figure S1. Histograms of Lipid Profile Among Healthy Individuals.

TC, total cholesterol; TG, triglyceride; HDL-C, high density lipoprotein cholesterol; LDL-C, low density lipoprotein cholesterol; VLDL-C, very low density lipoprotein cholesterol; Remnant-C, remnant-cholesterol.

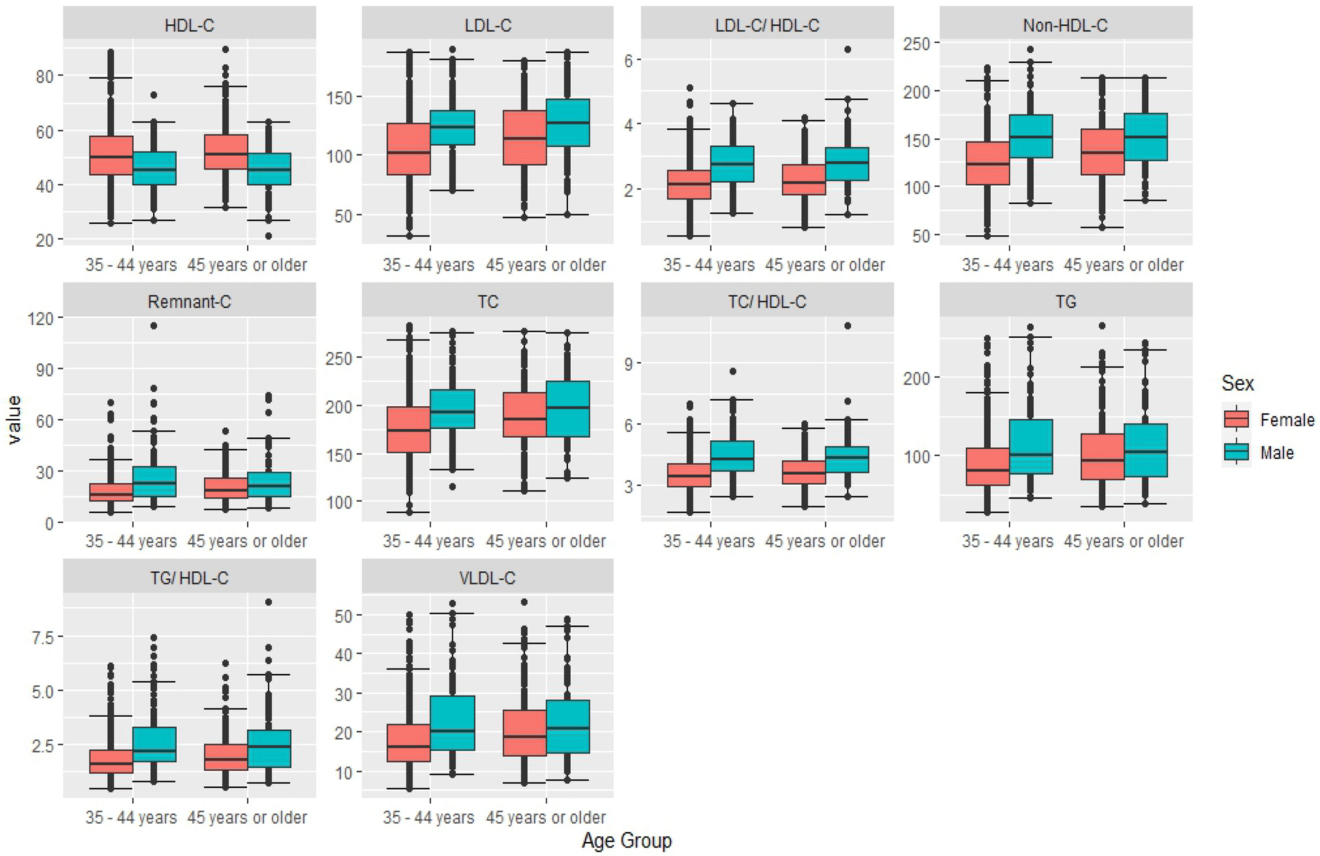

Figure S2. Distribution of Lipid Profile with Error Bars in Healthy People Based on Sex and Age Group.

TC, total cholesterol; TG, triglyceride; HDL-C, high density lipoprotein cholesterol; LDL-C, low density lipoprotein cholesterol; VLDL-C, very low density lipoprotein cholesterol; Remnant-C, remnant-cholesterol.
